# Whole Genome Sequencing Based Surveillance of *L. monocytogenes* for Early Detection and Investigations of Listeriosis Outbreaks

**DOI:** 10.3389/fpubh.2019.00139

**Published:** 2019-06-04

**Authors:** Ariane Pietzka, Franz Allerberger, Andrea Murer, Anna Lennkh, Anna Stöger, Adriana Cabal Rosel, Steliana Huhulescu, Sabine Maritschnik, Burkhard Springer, Sarah Lepuschitz, Werner Ruppitsch, Daniela Schmid

**Affiliations:** ^1^AGES - Austrian Agency for Health and Food Safety, Graz, Austria; ^2^AGES - Austrian Agency for Health and Food Safety, Vienna, Austria; ^3^European Public Health Microbiology training programme (EUPHEM), European Centre for Disease Prevention and Control (ECDC), Stockholm, Sweden

**Keywords:** whole-genome sequencing, pulsed-field gel electrophoresis, outbreak investigation, public health laboratory capacity, public health surveillance

## Abstract

In Austria, all laboratories are legally obligated to forward human and food/environmental *L. monocytogenes* isolates to the National Reference Laboratory/Center (NRL) for *Listeria*. Two invasive human isolates of *L. monocytogenes* serotype 1/2a of the same pulsed-field gel electrophoresis (PFGE) pattern, previously unknown in Austria, were cultured for the first time in January 2016. Five further human isolates, obtained from patients with invasive listeriosis between April 2016 and September 2017, showed this PFGE pattern. In Austria the NRL started to use whole-genome sequencing (WGS) based typing in 2016, using a core genome MLST (cgMLST) scheme developed by Ruppitsch et al. 2015, which contains 1701 target genes. Sequence data are submitted to a publicly available nomenclature server (Ridom GmbH, Münster, Germany) for allocation of the core genome complex type (CT). The seven invasive human isolates differed from each other with zero to two alleles and were allocated to CT1234 (declared as outbreak strain). Among the Austrian strain collection of about 6,000 cgMLST-characterized non-human isolates (i.e., food/environmental isolates) 90 isolates shared CT1234. Out of these, 83 isolates were traced back to one meat processing-company. They differed from the outbreak strain by up to seven alleles; one isolate originated from the company's industrial slicer. The remaining seven CT1234-isolates were obtained from food products of four other companies (five fish-products, one ready-to-eat dumpling and one deer-meat) and differed from the outbreak strain by six to eleven alleles. The outbreak described shows the considerable potential of WGS to identify the source of a listeriosis outbreak. Compared to PFGE analysis, WGS-based typing has higher discriminatory power, yields better data accuracy, and allows higher laboratory through-put at lower cost. Utilization of WGS-based typing results of human and food/ environmental *L. monocytogenes* isolates by appropriate public health analysts and epidemiologists is indispensable to support a successful outbreak investigation.

## Introduction

Listeriosis is a relatively uncommon disease, which typically causes a severe disease in a high portion of cases and deaths in susceptible population subgroups (1, 2). Listeriosis is a foodborne illness of major public health concern because of the severity of its complications (infections of the central nervous system, septicemia, gastroenteritis and abortion), a hospitalization rate of 98.6% and a case-fatality ratio of 13,8%, as reported by the EU summary report on zoonoses, zoonotic agents and food-borne outbreaks from 2017 (3). The surveillance of listeriosis in the European Union/European Economic Area (EU/EEA) focuses on the severe invasive forms of the disease for which the risk groups are mainly elderly and immunocompromised persons, pregnant women and infants. In 2017, 2,480 confirmed cases of invasive listeriosis were reported by 28 EU/EEA countries, resulting in an overall notification rate of 0.48 per 100,000 population (3). The increasing trend in the number of listeriosis cases in the EU/EEA, probably also due to the increased population size of the elderlies (4, 5), is worrying and calls for utmost attention to be placed on the prevention and control of the disease and outbreaks. The European Center for Disease Prevention and Control (ECDC), the European Food Safety Authority (EFSA) and the European Union Reference Laboratory (EU-RL) for *L. monocytogenes* have set up a joint database collecting, on a voluntary basis, combined AscI/ApaI PFGE profiles for PFGE typing data for human, food, animal and environmental isolates from public heath institutes and food safety and veterinary authorities to enable detection of listeriosis outbreaks affecting several countries (6). However, technical development is evolving fast and whole genome sequencing (WGS)-based typing methods replaced pulsed-field gel electrophoresis (PFGE) as the gold-standard showing higher accuracy and a superior discriminatory power (7). The outbreak described here, illustrates impressively the considerable potential of WGS based typing to elucidate the source of a listeriosis outbreak.

## Background to Outbreak Investigation

In Austria, laboratories have a legal obligation to forward human and food/environmental *L. monocytogenes* isolates derived from official controls as well as from ownchecks to the NRL. In January 2016, two human isolates of *L. monocytogenes* serotype 1/2a of the identical pulsed-field gel electrophoresis (PFGE) pattern, previously unknown in Austria, were cultured for the first time. Two environmental isolates of unknown origin, were obtained in January and February 2016, and another 24 food/environmental isolates were obtained between September 2016 and December 2017. In addition five further human isolates from patients with invasive listeriosis, isolated between April 2016 and September 2017 were obtained. All isolates showed this new PFGE pattern. The food and environmental isolates originated from six different laboratories. From January to August 2018, further 69 food/environmental isolates possibly related to the outbreak were sent to the National Reference Laboratory (NRL) for *Listeria*. In summary, a total of 95 non-human isolates together and seven human outbreak isolates were typed by using WGS cgMLST analysis. No reliable information was available on patients' relevant food consumption.

On 25 January 2018, Austria launched an Urgent Inquiry (UI-460) in The Epidemic Intelligence Information System (EPIS), a web-based communication platform that allows nominated public health experts to exchange technical information to assess whether current and emerging public health threats have a potential impact in the European Union. Aim of the outbreak investigation was to identify the source(s) and to recommend the appropriate public health measures for preventing further cases. Thirteen countries (Denmark, Finland, France, Germany, Ireland, Italy, Luxembourg, The Netherlands, Norway, Slovenia, Spain, Sweden and the United Kingdom) answered via the platform and eight countries reported cases with at least six allelic differences to the Austrian outbreak cluster. Raw data of the sequences were provided from the countries, which allowed a direct comparison with the Austrian database. Eight non-human strains isolated in France and the Netherlands were reported in the European Union Reference Laboratory for *Listeria monocytogenes* technical report [EURL Lm 2018 (8)] to form a cgMLST cluster with five to seven pairwise allele differences against the outbreak strain.

## Materials and Methods

### Origin of Isolates, Cultivation, and Genomic DNA Isolation

In Austria, *Listeria* isolates obtained from food and environmental samples, as well as human isolates, must be sent to the NRL for Listeria by legislation. The non-human isolates are anonymized, provided with unique identifier and information on the type of food matrix only (e.g., meat-product, diary-product, vegetable-product, food-environment) by the sending primary food laboratories. Isolates are cultivated on RAPID'L.Mono™ agar plates (Biorad, Munich, Germany) for species confirmation and subsequently subcultured overnight on Columbia Broth (BD Difco™, Heidelberg, Germany) for extraction of high molecular weight genomic DNA using the HMW MagAttract kit (Qiagen, Hilden, Germany) according to the instructions of the manufacturer for Gram positive bacteria.

### Whole Genome Sequencing and Data Analysis

Whole genome sequencing was performed as described previously (9). Briefly, for sequencing, an Illumina MiSeq platform (Illumina Inc., San Diego, CA, USA) was used. Library preparation was carried out using Nextera XT according to the instructions of the manufacturer (Illumina Inc., San Diego, CA, USA). For assembly into draft genomes, raw reads were *de novo* assembled using SPAdes version 3.11.1 (10). Contigs were filtered for a minimum coverage of 5-fold and minimum length of 200 bp, which resulted in 26–187 contigs at a coverage of 46–148-fold. Classical multilocus sequence typing (MLST) data according to Ragon et al. (11) and genoserotyping data according to Hyden et al. (12) were *de novo* extracted from WGS sequence data. Assessment of the core genome multilocus sequence typing (cgMLST) results was done using Ridom SeqSphere+ software version 5.1.0 as described by Ruppitsch et al. (13). All isolates had 98.1–99.8% good targets and a minimum spanning tree (MST) was generated in Ridom SeqSphere+ version 5.1.0 for visualization of strain relatedness. For comparison and data harmonization SeqSphere+ results were compared to the Pasteur cgMLST scheme (7) and GenomeGraphR (14). The sequences have been deposited in DDBJ/EMBL/GenBank under the project number PRJNA434392. Raw sequence data for each strain were deposited under SRA accession numbers (Table 1).

**Table 1:** Accession numbers of sequences available at NCBI Sequence Read Archive (SRA).

**ID**	**Accession no**.	**cgMLST**	**MLST CC**	**Genoserotype**
5F_CoA	SRR6740436	1234	155	IIa
6F_CoA	SRR6740437	1234	155	IIa
3E_CoA	SRR6740438	1234	155	IIa
4E_CoA	SRR6740439	1234	155	IIa
1E_uk	SRR6740440	1234	155	IIa
2E_uk	SRR6740441	1234	155	IIa
7H	SRR6740442	1234	155	IIa
4F_CoA	SRR6740443	1234	155	IIa
1F_CoA	SRR6740444	1234	155	IIa
2F_CoA	SRR6740445	1234	155	IIa
15F_CoA	SRR6740446	1234	155	IIa
16F_CoA	SRR6740447	1234	155	IIa
9F_CoA	SRR6740448	1234	155	IIa
10F_CoA	SRR6740449	1234	155	IIa
7F_CoA	SRR6740450	1234	155	IIa
8F_CoA	SRR6740451	1234	155	IIa
13F_CoA	SRR6740452	1234	155	IIa
14F_CoA	SRR6740453	1234	155	IIa
11F_CoA	SRR6740454	1234	155	IIa
12F_CoA	SRR6740455	1234	155	IIa
3F_CoA	SRR6740456	1234	155	IIa
28F_CoA	SRR6740457	1234	155	IIa
19F_CoA	SRR6740458	1234	155	IIa
18F_CoA	SRR6740459	1234	155	IIa
17F_CoA	SRR6740460	1234	155	IIa
4H	SRR6740461	1234	155	IIa
3H	SRR6740462	1234	155	IIa
2H	SRR6740463	1234	155	IIa
1H	SRR6740464	1234	155	IIa
6H	SRR6740465	1234	155	IIa
5H	SRR6740466	1234	155	IIa
90F_CoA	SRR8184623	6743	37	IIa
56F_CoA	SRR8185109	1234	155	IIa
55F_CoA	SRR8185110	1234	155	IIa
58F_CoA	SRR8185111	1234	155	IIa
57F_CoA	SRR8185112	1234	155	IIa
52F_CoA	SRR8185113	1234	155	IIa
51F_CoA	SRR8185114	1234	155	IIa
40F_CoA	SRR8185115	1234	155	IIa
39F_CoA	SRR8185116	1234	155	IIa
38F_CoA	SRR8185117	1234	155	IIa
37F_CoA	SRR8185118	1234	155	IIa
36F_CoA	SRR8185119	1234	155	IIa
35F_CoA	SRR8185120	1234	155	IIa
34F_CoA	SRR8185121	1234	155	IIa
33F_CoA	SRR8185122	1234	155	IIa
32F_CoA	SRR8185123	1234	155	IIa
31F_CoA	SRR8185124	1234	155	IIa
72F_CoA	SRR8185125	1234	155	IIa
71F_CoA	SRR8185126	1234	155	IIa
74F_CoA	SRR8185127	1234	155	IIa
78F_CoA	SRR8185128	1234	155	IIa
76F_CoA	SRR8185129	1234	155	IIa
27F_nonCoA	SRR8185130	1234	155	IIa
77F_CoA	SRR8185131	1234	155	IIa
73F_CoA	SRR8185132	1234	155	IIa
54F_CoA	SRR8185133	1234	155	IIa
53F_CoA	SRR8185134	1234	155	IIa
89F_CoA	SRR8185135	1234	155	IIa
69F_CoA	SRR8185136	1234	155	IIa
70F_CoA	SRR8185137	1234	155	IIa
65F_CoA	SRR8185138	1234	155	IIa
66F_CoA	SRR8185139	1234	155	IIa
67F_CoA	SRR8185140	1234	155	IIa
68F_CoA	SRR8185141	1234	155	IIa
61F_CoA	SRR8185142	1234	155	IIa
62F_CoA	SRR8185143	1234	155	IIa
63F_CoA	SRR8185144	1234	155	IIa
64F_CoA	SRR8185145	1234	155	IIa
47F_CoA	SRR8185146	1234	155	IIa
48F_CoA	SRR8185147	1234	155	IIa
45F_CoA	SRR8185148	1234	155	IIa
46F_CoA	SRR8185149	1234	155	IIa
43F_CoA	SRR8185150	1234	155	IIa
44F_CoA	SRR8185151	1234	155	IIa
41F_CoA	SRR8185152	1234	155	IIa
42F_CoA	SRR8185153	1234	155	IIa
60F_CoA	SRR8185154	1234	155	IIa
49F_CoA	SRR8185155	1234	155	IIa
50F_CoA	SRR8185156	1234	155	IIa
59F_CoA	SRR8185157	1234	155	IIa
83F_CoA	SRR8185158	5753	517	IIb
29F_CoA	SRR8185159	6252	155	IIa
25F_nonCoA	SRR8185160	1234	155	IIa
24F_nonCoA	SRR8185161	1234	155	IIa
75F_CoA	SRR8185162	1234	155	IIa
26F_nonCoA	SRR8185163	1234	155	IIa
21F_nonCoA	SRR8185164	1234	155	IIa
5E_CoA	SRR8185165	1234	155	IIa
23F_nonCoA	SRR8185166	1234	155	IIa
22F_nonCoA	SRR8185167	1234	155	IIa
81F_CoA	SRR8185168	6399	451	IIa
82F_CoA	SRR8185169	6424	1	IVb
87F_CoA	SRR8185170	1234	155	IIa
84F_CoA	SRR8185171	1234	155	IIa
79F_CoA	SRR8185172	1234	155	IIa
88F_CoA	SRR8185173	1234	155	IIa
80F_CoA	SRR8185174	1234	155	IIa
85F_CoA	SRR8185175	1234	155	IIa
30F_CoA	SRR8185176	1234	155	IIa
86F_CoA	SRR8185177	1234	155	IIa
20F-CoA	SRR8186973	1234	155	IIa

*BioProject ID: PRJNA434392; Title: LIST_2018_CT1234. E_uk, environmental isolate of unknown origin (at the time of submission); E_CoA, environmental isolate, company A-associated; F_CoA, food-isolate, company A-associated; H, human isolate*.

SNP analysis was done with GenomeGraphR Beta 2.7 [Sanaa et al. (14)] using the default settings and a cluster threshold definition of 12 SNPs. All strains were compared with the isolates present in the database.

## Results

Austria reported a suspected outbreak due *to L. monocytogenes* serotype 1/2a of the same PFGE pattern, including seven patients of invasive listeriosis, having occurred in eastern Austria between 2015 and 2017. The cgMLST typing of the seven human invasive isolates revealed a genetically tight cluster, complex type 1234 (CT1234), which corresponds to CT1170 of Institut Pasteur cgMLST scheme [Moura et al. (7)], with zero to two allelic differences from each other. SNP analysis revealed that our clinical isolates differed from each other by 1–4 SNPs. In addition, the closest clinical strain clustering with our isolates differed by 11–12 SNPs and therefore confirmed the CT1234.

On 26 January 2018, the Austrian Ministry of Health mandated the Austrian Agency for Health and Food Safety (AGES) to investigate this suspected outbreak. A confirmed outbreak case was defined as a patient with invasive listeriosis, positive for *L. monocytogenes* cgMLST CT1234 isolate, which differed by ≤ 2 alleles from a representative outbreak isolate by using cgMLST, and with a disease onset on or after 1 January 2015.

**Figure 1:**
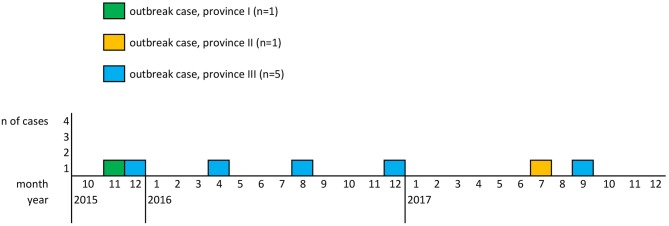
Outbreak cases of *L. monocytogenes* CT1234 by month of disease onset and province of residence, 2015–2017, Austria.

The aforementioned seven patients fulfilled the definition of a confirmed outbreak case. Patients were 29–97 years old (mean: 68; median: 73), five females and two males, with disease onset between November 2015 and September 2017 and residence in three of the nine Austrian provinces. Figure 1 depicts the outbreak cases by month of diagnosis and province of residence.

Among the Austrian genome database of about 6,000 non-human isolates (i.e., food/environmental isolates, collected between 2015 and 2018), 90 isolates shared genoserotype IIa, MLST CC155, and cgMLST CT1234. Out of these, 83 isolates were traced back to a meat-processing company (companyA; CoA) in eastern Austria. These food/environmental isolates differed from the outbreak strain by zero to seven alleles and one isolate, originated from the company's industrial slicer. The remaining seven CT1234 isolates were obtained from food products of four other companies (five fish-products, one ready-to-eat dumpling and deer-meat product each) and differed from the main outbreak strain by six to eleven alleles.

**Figure 2:**
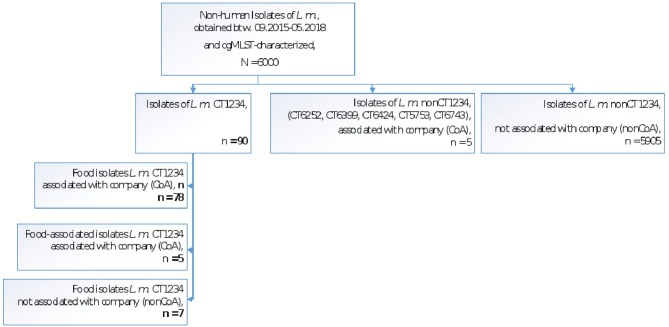
Non-human *L. monocytogenes* isolates of the AGES strain collection (*L.m*. CT1234, *L.m*. nonCT1234) with and without company A linkage (CoA, nonCoA).

Figure 2 illustrates the non-human isolates of the AGES *Listeria* strain collection by CT1234 allocation and linkage to the meat processing company A.

**Figure 3:**
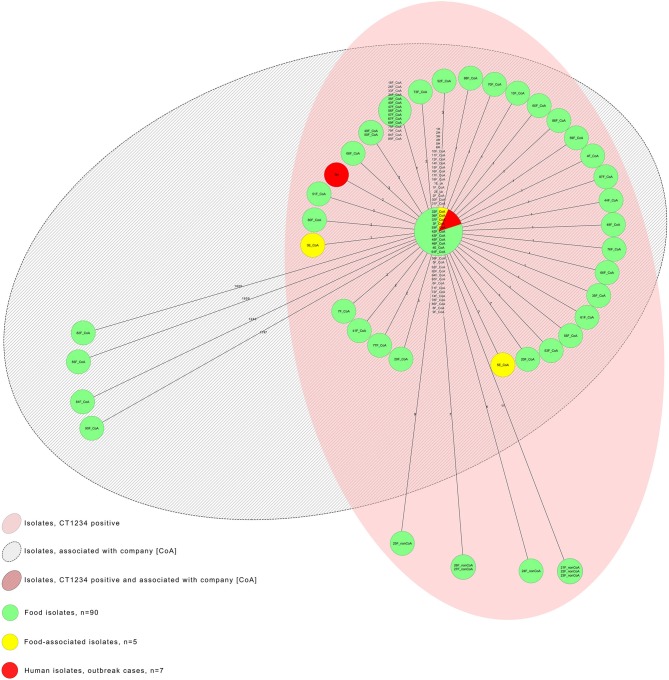
Adapted minimum spanning tree of the seven human outbreak isolates, the food and environmental isolates of *L. monocytogenes*, including the company A associated and non-associated CT1234-isolates (CoA CT1234-isolates and nonCoA CT1234-isolates; *n* = 90), and the five company A associated nonCT1234-isolates; *N* = 102.

Figure 3 illustrates the MST of the seven human outbreak isolates, of food and environmental isolates of *L. monocytogenes*, comprising company A associated and non-associated CT1234-isolates (CoA, nonCoA), and company A associated, nonCT1234-isolates.

In November 2017, a 47-year old male developed signs and symptoms compatible with a non-invasive listeriosis (i.e., febrile gastroenteritis) 7 h after consumption of a pizza with sliced ham topping in a restaurant in the Austrian province Tyrol. No patient isolate of *L.monocytogenes* was available. The official sample taken from the sliced pizza ham at the restaurant tested positive for *L. monocytogenes* CT1234 with one allelic difference from the outbreak strain (Figure 3: 28F_CoA). Trace-back analyses identified the origin of the ham pizza topping from meat-processing company A.

### Public Health Measures

Company A implemented control measures including intensified environmental disinfection, installation of a new slicer and continuous investigation of environmental swabs and newly processed food products for *Listeria*. All food batches had to be negative for *L. monocytogenes* before being released to the market. During these activities, further four strains of *L. monocytogenes* were found in the tested food products of the company. There were of complex types CT6252 (genoserotype IIa, MLST CC155), CT6399 (genoserotype IIa, MLST CC451), CT6424 (genoserotype IVb, MLST CC1), CT6743 (genoserotype IIa, MLST CC37), different from the outbreak CT, CT1234 (Figure 3: food isolates 81F, 82F, 83F, and 90F). After August 2018, the public health authorities found no further *L. monocytogenes* positive food products.

## Discussion

Investigation of listeriosis outbreaks is difficult due to the multitude of possible food vehicles including a broad range of ready-to-eat foods. Pulsed-field gel electrophoresis (PFGE) was the gold standard for strain typing (6) but has become obsolete with the advent of WGS. WGS is highly discriminatory and superior for allocating listeriosis cases to an outbreak (7, 13). Due to this superiority of WGS it is time to stop PFGE (Pulsenet Network resolution, ECDC). A dictionary between PFGE and MLST will allow to screen the PFGE database for previous strains using WGS specific ST or CC (15). However, with the limitations of current WGS technology we cannot create PFGE patterns from WGS data and therefore cannot create a PFGE-WGS dictionary. Despite the availability of technical literature on methods for outbreak investigations, there are no pre-specified formulae to dictate the path that an outbreak investigation is supposed to take (16). Investigations of listeriosis outbreaks provide a unique opportunity to gain new scientific knowledge on the occurrence of *L. monocytogenes* in the food-processing setting.

In contrast to Europe, the United States have a zero tolerance policy for *L. monocytogenes* in ready-to-eat foods (17). Commission Regulation (EC) No 2073/2005 of 15 November 2005 on microbiological criteria for foodstuffs requires a limitation of < 100 CFU/g in ready-to-eat food products able to support the growth of *L. monocytogenes* (other than those intended for infants and for special medical purposes) during the shelf life, when products are placed on the market; these particular food products must be tested for the absence of *L. monocytogenes* in 25 g before leaving the immediate control of the producing food business operator. Otherwise a challenge test which ensures that the limit of 100 cfu/g at the end of shelf-life will not be exceeded, has to be shown (18). Although the infective dose of *L. monocytogenes* is unknown and population subgroups differ in vulnerability to *Listeria monocytogenes* in food, the present European legislation should be sufficient in controlling foodborne listeriosis.

As a consequence of an earlier outbreak of listeriosis in Austria (19, 20), the Federal Ministry of Health had classified 600 food-producing facilities as being at high risk of *Listeria* contamination and ordered the provinces to conduct inspections on various control measures in a Key Activity Action Campaign entitled “Schwerpunktaktion SPA-A-600” in 2014. The province to which company A was assigned had neglected to complete this requirement. From 2015 to 2017, only one official sample of sliced bacon was obtained on 22 November 2017 by the local food authority at the meat processing company A. The final report, outlining the presence of *L. monocytogenes* in numbers below 100 CFU/g, was not issued until 1 February 2018. Surprisingly, no challenge test was performed for this food-product, especially considering that a similar outbreak caused by the consumption of bacon that was contaminated with *L. monocytogenes* caused four fatalities in Bavaria. At the time in 2016, this outbreak led to a public recall and public warning in Austria (21).

For two decades, PFGE was the reference method for *L. monocytogenes* surveillance and outbreak investigation (2, 22). It is still used for screening but is increasingly replaced by WGS based typing methods (7, 14, 21, 23–26). WGS based typing outperforms PFGE with respect to the discriminatory power, information content, throughput, reproducibility, costs and inter-laboratory data exchange. However, it is important to keep in mind that the differences between the cgMLST schemes of Moura et al. and Ruppitsch et al. can have an impact on the cluster detection (24). For communication on detected clusters it is important to know which core genome scheme, assembler, and assembler version and sequencing technology was used and which average sequencing coverage was achieved.

Based on the current cgMLST analysis of the human outbreak isolates, a difference from each other by only zero to two alleles, and of the majority of the outbreak associated food-isolates, a difference by zero to four alleles should be considered to increase the specificity of linking isolates to *L. monocytogenes* outbreaks. WGS data not only allow to infer phylogenetic relationships but also to filter for additional information like serotypes (12), virulence- and resistance-genes (7).

Although it is known that SNP analysis provides maximal discriminatory power, results are difficult to standardize and interpret (27). Moreover, the analysis based on single nucleotide polymorphisms (SNPs) showed here identical results to the ones obtained by cgMLST. Expansion of the classical MLST principle to a genome wide gene-by-gene comparison allowed the establishment of databases based on well-defined core genome or whole genome MLST schemes (7, 13, 24, 28). The setup of open accessible databases (*Listeria monocytogenes* cgMLST at https://www.cgmlst.org/ncs/schema/690488/, BIGSdb-*Lm* at http://bigsdb.pasteur.fr/listeria) allows the comparison and sharing of data between public health laboratories worldwide and facilitates international source tracking and multinational outbreak investigation (29, 30). These new WGS databases, although only 3 years old, already harbor nearly twice the number of strain complex types (CT) than the >20 year old PulseNet PFGE database demonstrating again the higher discriminative power of WGS based typing. Compared to PFGE, these major improvements in *L. monocytogenes* typing allow a faster and more discriminative detection of clusters and reduce unnecessary epidemiological investigations. *L. monocytogenes* is one of the pathogens for which a rapid transition from traditional typing methods to WGS-based typing methods is presently occurring in the public health laboratories of the EU/EEA as well as the PulseNet International network, and it is the first food- and waterborne pathogen for which a comprehensive WGS-assisted real-time surveillance is planned to be established at the EU/EEA level (31). Due to the superiority of WGS for real-time surveillance in a One Health approach, the PulseNet International network and EU/EEA health and food safety authorities move to cgMLST and wgMLST analysis (24, 30).

## Conclusions

Compared to PFGE analysis, WGS based typing has a higher discriminatory power, yields better data accuracy, and allows higher laboratory through-put at lower cost, as proven in the current outbreak investigation (26). The meaningful use of WGS based typing data for a successful investigation of a listeriosis outbreak and the appropriate public health measures, requires intense collaboration between the public health and food safety authorities, food microbiologists, typing experts and epidemiologists.

## Author Contributions

AP, DS, FA, and WR contributed conception and design of the study, data analysis and interpretation and writing of the manuscript. AM, AL, AS, AC, SM, and SL performed data analysis. SH and BS contributed to the interpretation of data of the work and critically revised the content of the study. All authors contributed to manuscript revision, read and approved the submitted version.

### Conflict of Interest Statement

We declare that none of the authors have any commercial and financial relationship to the company Ridom GmbH (Münster, Germany), developer of the Ridom SeqSphere+ software, mentioned in the manuscript. The authors declare that the research was conducted in the absence of any commercial or financial relationships that could be construed as a potential conflict of interest.
